# Perforated duodenal ulcer presenting with a subphrenic abscess revealed by plain abdominal X-ray films and confirmed by multi-detector computed tomography: a case report

**DOI:** 10.1186/1752-1947-7-257

**Published:** 2013-11-11

**Authors:** Luigi Camera, Milena Calabrese, Valeria Romeo, Fabrizio Scordino, Pier Paolo Mainenti, Marco Clemente, Gaetano Rapicano, Marco Salvatore

**Affiliations:** 1 Department of Radiology, University ‘Federico II’, Via S. Pansini 5, 80131 Naples, Italy; 2 Institute of Biostructures and Bioimaging, National Research Council (C.N.R.), Via Tommaso De Amicis 95, 80145 Naples, Italy; 3 Department of Infectious Diseases, University ‘Federico II’, Via S. Pansini 5, 80131 Naples, Italy; 4 Department of Emergency Surgery, AORN ‘A. Cardarelli’, Naples, Italy

**Keywords:** Peptic ulcer disease, Subphrenic abscess, Abdominal plain film, Multi-detector computed tomography

## Abstract

**Introduction:**

Peptic ulcer disease is still the major cause of gastrointestinal perforation despite major improvements in both diagnostic and therapeutic strategies. While the diagnosis of a perforated ulcer is straightforward in typical cases, its clinical onset may be subtle because of comorbidities and/or concurrent therapies.

**Case presentation:**

We report the case of a 53-year-old Caucasian man with a history of chronic myeloid leukemia on maintenance therapy (100mg/day) with imatinib who was found to have a subphrenic abscess resulting from a perforated duodenal ulcer that had been clinically overlooked. Our patient was febrile (38.5°C) with abdominal tenderness and hypoactive bowel sounds. On the abdominal plain X-ray films, a right subphrenic abscess could be seen. On contrast-enhanced multi-detector computed tomography, a huge air-fluid collection extending from the subphrenic to the subhepatic anterior space was observed. After oral administration of 500cm^3^ of 3 percent diluted diatrizoate meglumine, an extraluminal leakage of the water-soluble iodinated contrast media could then be appreciated as a result of a perforated duodenal ulcer. During surgery, the abscess was drained and extensive adhesiolysis had to be performed to expose the duodenal bulb where the ulcer was first identified by methylene blue administration and then sutured.

**Conclusions:**

While subphrenic abscesses are well known complications of perforated gastric or duodenal ulcers, they have nowadays become rare thanks to advances in both diagnostic and therapeutic strategies for peptic ulcer disease. However, when peptic ulcer disease is not clinically suspected, the contribution of imaging may be substantial.

## Introduction

Peptic ulcer disease is still the major cause of gastrointestinal perforation, despite major improvements in both diagnostic and therapeutic strategies [1].

The diagnosis of a perforated ulcer is straightforward when an acute onset of epigastric pain is observed in a patient with a known history of peptic ulcer disease [2]. In such instances, radiological investigation is usually limited to plain abdominal X-ray films to document the associated pneumoperitoneum [3].

Less commonly, clinical onset of a perforated gastric or duodenal ulcer may be atypical [4] or subtle because of comorbidities [5] and/or concurrent therapies [6]. In such cases, the contribution of imaging may be substantial [7-12]. Indeed, computed tomography (CT) has been established as the most valuable imaging technique for identifying the presence, site and cause of gastrointestinal tract perforation, and this is particularly true since the advent of multi-detector CT (MDCT) technology [9-12].

Here, we report the case of a 53-year-old man with chronic myeloid leukemia who was found to have a huge subphrenic abscess due to a perforated duodenal ulcer, which had been clinically overlooked for almost two weeks.

## Case presentation

A 53-year-old Caucasian man with a history of chronic myeloid leukemia in clinical remission for three years, on maintenance therapy (100mg/day) with imatinib, was admitted to our hospital to investigate a persistent fever. He reported the sudden onset of an acute chest pain with epigastric radiation 15 days before his hospital admission. At that time, the referring physician excluded a pain of cardiac origin based on normal electrocardiogram (ECG) findings and cardiac enzyme levels. Three days later, our patient was febrile (38.5°C) and dyspnoic. Laboratory tests revealed an elevated white blood cell count (13×10^3^ cells/mL), and a chest X-ray (not shown) was performed revealing an ill-defined hypolucency in the right lower lobe. On the basis of these clinical and radiological findings, a presumed diagnosis of acute bronchopneumonia was made and our patient was given medical therapy with antibiotics, non-steroidal anti-inflammatory and proton-inhibitor drugs.

**Figure 1:**
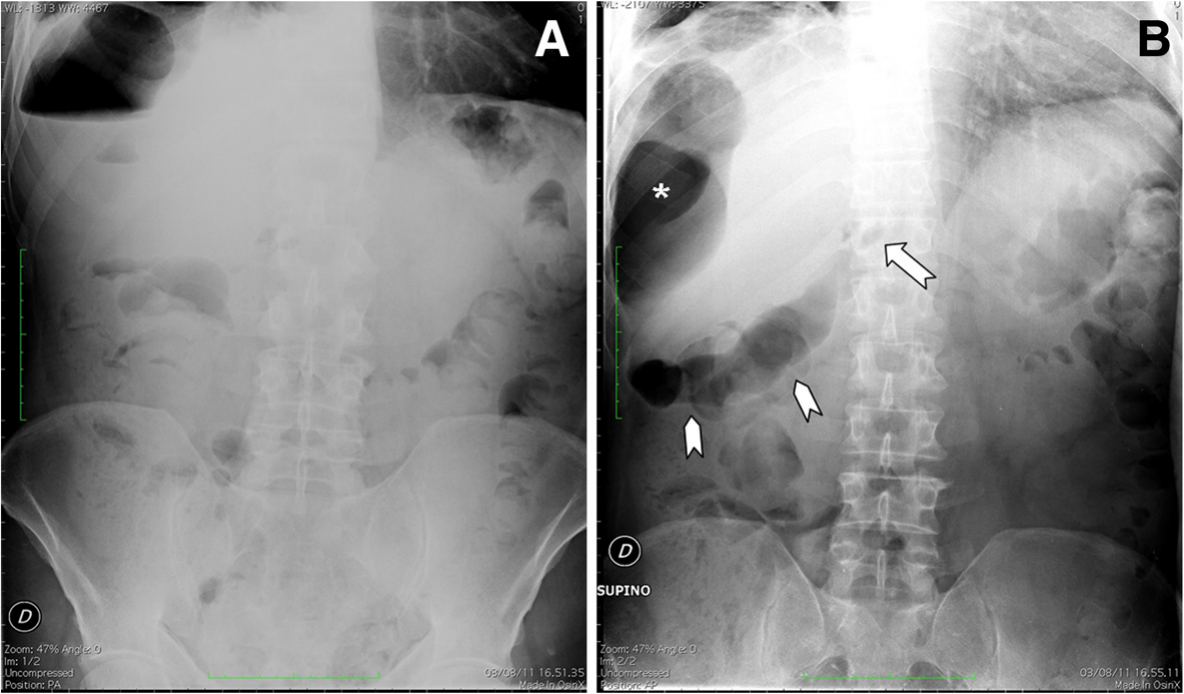
**Abdominal plain X-ray films obtained in the upright (A) and supine position (B).** In **(A)** a huge air-fluid level can be seen in the right subphrenic space. In **(B)** the extraluminal air appears to extend from the perihepatic (asterisk) to the subhepatic space (arrow-heads). Extraluminal air can also be appreciated in the hepatoduodenal fossa (arrow) pinpointing the perforated duodenal ulcer.

At admission, our patient was febrile (38.5°C) with abdominal tenderness and hypoactive bowel sounds. A plain abdominal X-ray film was then performed (Figure 1). On the upright film (Figure 1A), a huge air-fluid level was clearly depicted in the right subphrenic space. In the supine position the extraluminal air appear to extend from the right subphrenic to the subhepatic space and the hepatoduodenal fossa (Figure 1B). Based on the X-ray findings, a contrast-enhanced multi-detector row CT study was performed (Aquilion 64; Toshiba, Tokyo, Japan) with a detector configuration of 1×32mm, a table feed of 36mm/s and a gantry rotation time of 0.75 (pitch factor=0.844), 120kVp and automatic dose modulation. A monophasic acquisition was performed 80s after intravenous bolus injection of 150cm^3^ of non-ionic iodinated contrast media (Ultravist 370; Bayer, Berlin, Germany) at a rate of 2cm^3^/s.

**Figure 2:**
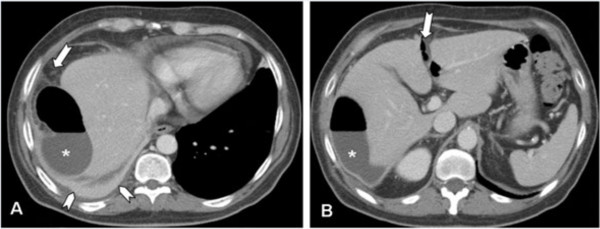
**Multi-detector contrast-enhanced computed tomography.** Axial scans at the level of the upper abdomen are shown. In **(A)** a huge air-fluid collection (asterisk) can be seen in the right subphrenic space with mild stranding of the surrounding fat (arrow). There are also reactive pericardial and pleural effusions, the latter with associated atelectasia of the right lung base (arrowheads). In **(B)** the air-fluid collection (asterisk) appears to extend to the perihepatic space. Extraluminal air bubbles can also be detected in the fissure of Teres’ ligament (arrow).

**Figure 3:**
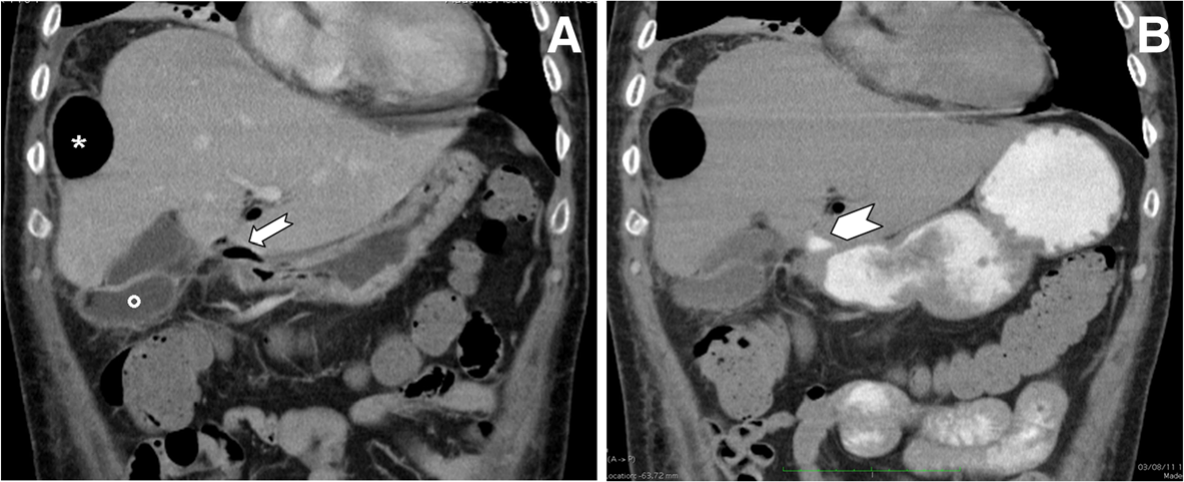
**Multi-detector contrast-enhanced computed tomography.** Coronal reformatted images obtained before **(A)** and after **(B)** oral administration of 500cm^3^of 3 percent diluted diatrizoate meglumine are shown. In **(A)** extraluminal air can be seen in the perihepatic space (asterisk) as well as in the hepatoduodenal ligament (arrow). The fluid component of the abscess (circle) can also be detected beside the gallbladder. In **(B)** the extraluminal leakage of the water-soluble iodinated contrast media can be well appreciated at the level of the hepatoduodenal ligament (arrowhead) in place of the extraluminal air.

On contrast-enhanced MDCT, a right subphrenic abscess was clearly depicted (Figure 2A,B). The huge air-fluid collection extended from the subphrenic (Figure 2A) to the perihepatic space (Figure 2B) and extraluminal air bubbles could also be detected in the fissure of Teres’ ligament (Figure 2B). On the coronal reformatted image extraluminal air could also be detected in the hepatoduodenal ligament (Figure 3A) where extraluminal leakage of the water-soluble iodinated contrast media could be seen (Figure 3B) after oral administration of 500cm^3^of 3 percent diluted diatrizoate meglumine.

Our patient underwent surgery. During laparotomy, a huge abscess was found in both the right subphrenic and the subhepatic spaces. After drainage, several attempts to identify the site of leakage were made but were unsuccessful because of an inflammatory block involving the lesser omentum, the duodenal bulb, the hepatic flexure and the inferior margin of the left hepatic lobe. After extensive adhesiolysis, the duodenal bulb was finally exposed and the site of the ulcer identified by methylene blue administration through a nasogastric tube. The ulcer was sutured.

Our patient had a largely uneventful recovery, with the only incidents being a right pleural fluid collection (600cm^3^) requiring thoracocentesis and small (<200cm^3^) residual perihepatic fluid collections, which were monitored and managed conservatively. He was discharged 12 days later.

## Discussion

Despite recent improvements in both diagnostic and therapeutic strategies for peptic ulcer disease [1], perforated peptic ulcer still represents the major cause of gastrointestinal perforation and the second most common complication of peptic ulcer disease [2].

When a perforated peptic ulcer is clinically suspected it represents an emergent condition prompting immediate surgery [13]. However, the clinical onset of a perforated gastric or duodenal ulcer may be atypical [4] or the perforation may be clinically overlooked in the presence of comorbidities [5] or it can be masqueraded by concurrent therapies [6]. In our patient’s case, it can be argued that an anti-inflammatory effect was somehow induced by the multi-kinase inhibitor drug imatinib, which our patient had been taking daily for seven years. However, a direct gastrointestinal toxicity of tyrosine kinase inhibitors in patients with chronic myeloid leukemia has also been described [14]. As far as the misdiagnosis of acute bronchopneumonia is concerned, it was largely based on an erroneous interpretation of the abnormal chest X-ray findings (not shown). While the presence of basal pulmonary infiltrates and/or pleural effusion should be well recognized as an indirect evidence of a subdiaphragmatic infection [15], this was not appreciated in our patient’s case. As far as the missed diagnosis of the right subphrenic abscess is concerned, we can only argue that its air component had likely been mistaken for the hepatic flexure as in Chilaiditi’s syndrome, despite the absence of austral folds [16].

Regardless, whenever a perforated peptic ulcer is not clinically suspected the contribution of imaging studies may be substantial and the diagnostic role of CT is undisputed [7-12]. This is particularly true since the advent of multi-detector technology that allows isotropic data set acquisition resulting in high-resolution images on both the axial as well as the coronal and sagittal planes [9-12].

Using CT, diagnosis of alimentary tract perforation can be based on both direct [11,12] and indirect findings [7-10]. Aside from free intraperitoneal air, direct findings of gastrointestinal tract perforation include the evidence of discontinuation of the bowel wall and/or the leakage of water-soluble contrast material. The former is now facilitated by the use of thin slice collimations with coronal and sagittal reformations as in multi-detector CT [11,12]. As far as the leakage of water-soluble contrast material is concerned, it simply relies on oral administration of iodinated contrast media; however, this is considered a controversial practice in patients with a clinical suspicion of gastrointestinal tract perforation [7-12].

In our patient’s case, the diagnosis of perforated peptic ulcer was indeed based on the leakage of the iodinated contrast material at the level of the duodenal bulb (Figure 3B) although the evidence of extraluminal air close to the duodenal bulb (Figure 3A) could also have been considered highly suggestive of a perforated duodenal ulcer [10]. In the present case, however, oral administration of iodinated contrast material was deemed necessary to precisely identify the perforation site in view of a laparoscopic approach that nowadays represents the therapeutic option of choice even in the presence of an abscess [13]. Our patient, however, underwent an open laparotomy because of his poor clinical condition and despite the anatomic details provided by MDCT it was necessary to administer methylene blue through his nasogastric tube to identify the perforated ulcer. This was masked by an inflammatory block involving the duodenal bulb along with the lesser omentum, the hepatic flexure of the colon and the inferior margin of the left hepatic lobe.

While most gastroduodenal perforations will manifest on CT with either direct or indirect findings, there may be cases in which they cannot be detected [3]. In such cases, a self-sealed perforation site or a perforation contained by adjacent organs can be postulated [8].

More commonly, the perforation may be clinically silent and lead to the formation of abscesses in the peritoneal cavity. Indeed, abscesses were found in 12 out of 73 patients (16 percent) with gastrointestinal perforation [9].

In our patient’s case, the diagnosis of a subphrenic abscess was prompted by abnormal abdominal X-ray film findings (Figure 1) and then confirmed by contrast-enhanced MDCT (Figure 2). As far as the former are concerned, while the huge air-fluid level depicted in the right subphrenic space (Figure 1A) could be considered consistent with a subphrenic abscess [15], the supine film pointed to the correct diagnosis of a perforated duodenal ulcer since the extraluminal air could be traced back to the hepatoduodenal ligament through the subhepatic space (Figure 1B). However, since the diagnosis of duodenal ulcer was not even clinically suspected, a contrast-enhanced MDCT had to be performed.

## Conclusions

Here we report a case of a perforated duodenal ulcer complicated by a right subphrenic abscess, first revealed on abdominal X-ray film and then confirmed by contrast-enhanced MDCT. While subphrenic abscesses are well known complications of perforated gastric or duodenal ulcers, they have nowadays become rare thanks to advances in both diagnostic and therapeutic strategies for peptic ulcer disease. However, when peptic ulcer disease is not clinically suspected, the contribution of imaging may be substantial.

## Consent

Written informed consent was obtained from the patient for publication of this manuscript and any accompanying images. A copy of the written consent is available for review by the Editor-in-Chief of this journal.

## Competing interests

The authors declare that they have no competing interests.

## Authors’ contributions

LC observed our patient and revised the manuscript. MC was responsible for the drafting of the manuscript. VR performed the literature research. FS was the referring physician. PPM performed the literature research. MC performed the laparotomy procedure. GR performed the laparotomy procedure. MS was responsible for manuscript editing. All authors read and approved the final manuscript.
